# Adropin Is a Key Mediator of Hypoxia Induced Anti-Dipsogenic Effects via TRPV4-CamKK-AMPK Signaling in the Circumventricular Organs of Rats

**DOI:** 10.3389/fnmol.2017.00105

**Published:** 2017-04-20

**Authors:** Fan Yang, Li Zhou, Xu Qian, Dong Wang, Wen-Juan He, Zhong-wei Tang, Jun Yin, Qing-Yuan Huang

**Affiliations:** ^1^Department of Pathophysiology and High Altitude Pathology, College of High Altitude Military Medicine, Third Military Medical UniversityChongqing, China; ^2^Key Laboratory of High Altitude Medicine, Third Military Medical University, Ministry of EducationChongqing, China; ^3^Key Laboratory of High Altitude Medicine, PLA, Third Military Medical UniversityChongqing, China; ^4^Department of Pharmacy, Xinqiao Hospital and The Second Affiliated Hospital, The Third Military Medical UniversityChongqing, China; ^5^Ba Gong li Sanatorium, The Chinese People’s Liberation Army 77200 TroopsKunming, China

**Keywords:** adropin, water intake, TRPV4, CamKK, AMPK, phosphorylation

## Abstract

Water intake reduction (anti-dipsogenic effects) under hypoxia has been well established, but the underlying reason remains unknown. Our previous report indicated that activated TRPV4 neurons in SFO are associated with anti-dipsogenic effects under hypoxia. Although low partial pressure of blood oxygen directly activates TRPV4, humoral factors could also be involved. In the present study, we hypothesize that adropin, a new endogenous peptide hormone, was rapidly increased (serum and brain) concomitant with reduced water intake in early hypoxia. Also, the nuclear expression of c-Fos, a marker for neuronal activation, related to water-consumption (SFO and MnPO) was inhibited. These effects were mitigated by a scavenger, rat adropin neutralizing antibody, which effectively neutralized adropin under hypoxia. Interestingly, injection of recombinant adropin in the third ventricle of the rats also triggered anti-dipsogenic effects and reduced c-Fos positive cells in SFO, but these effects were absent when TRPV4 was knocked down by shRNA. Moreover, adropin-activated CamKK-AMPK signaling related to TRPV4 calcium channel in SFO in normoxia. These results revealed that dissociative adropin was elevated in acute hypoxia, which was responsible for anti-dipsogenic effects by altering TRPV4-CamKK-AMPK signaling in SFO.

## Introduction

The balance of body fluid homeostasis is fragile in high altitude environment. Insensible evaporation increases the body fluid loss in climbers, while hypoxia, the most crucial risk factor in the plateau, strongly suppresses water consumption (anti-dipsogenic effects), which can lead to dehydration (Westerterp, 2001). For instance, the accumulated water intake decreases more than 70% on the first day of hypoxic rats (Jones et al., 1981a,b; Yang F. et al., 2016). The appropriate water loss and blood concentration is conducive to increase the efficiency of red blood cells carrying oxygen, improving the ability of acclimatization, which may be very important for the early migratory population (Yang F. et al., 2016). In contrast, excessive suppression of thirst, can also cause more serious water metabolic disorders, such as dehydration and abnormal water distribution, which is related to deep vein thrombosis (Gupta and Ashraf, 2012; Hull et al., 2016) and high altitude pulmonary edema (Sawka et al., 2015; Hilty et al., 2016).

Although anti-dipsogenic effects were first noted in 1981 (Jones et al., 1981a,b), the underlying mechanism needs further elucidation. Various regions in the circumventricular organs (CVOs), including Subfornical organ (SFO), Median preoptic nucleus (MnPO) and Organum vasculosum of the lamina terminalis (OVLT), of the hypothalamus are activated in response to dehydration (McKinley et al., 2003). CVOs have few nuclei lacking in the blood-brain barrier (BBB) of the central nervous system (CNS), and are exposed to blood component (McKinley et al., 2003, 2006). In addition, intravenous injection of angiotensin, a vasoactive hormone that stimulates drinking, has been shown to activate CVO neurons in several species, and hyperosmotic stimulation of CVO nuclei increased fluid consumption in rats (Epstein et al., 1969; Sturgeon et al., 1973; Xu et al., 2015). Adropin, a recently discovered peptide hormone produced in the brain and liver, has pathologically relevant effects on energy homeostasis and lipogenesis catabolism, and exerts significant suppressive effects on thirst after intracranial injection (Stein et al., 2016). Hypoxia enhances catabolism, which may elevate serum adropin. For instance, increased serum adropin is often regarded as a candidate diagnostic marker for myocardial hypoxic-ischemic injury (Aydin et al., 2014; Yang C. et al., 2016). Our previous studies revealed that hypoxia activated TRPV calcium channel in CVOs, which was related to anti-dipsogenic effects. We hypothesis that if a humoral factor adropin that responds to acute hypoxia could be identified, it might control thirst and water consumption to adjust TRPV4 in the CVOs (Yang F. et al., 2016). In the present study, using c-Fos as a marker for neuronal activation, we examined the relationship between hypoxia, adropin and SFO neuronal activity, and uncovered the role of TRPV4-CamKK-AMPK signaling in the regulation of thirst.

## Materials and Methods

### Animals and Surgical Procedure

This study was performed in accordance with China’s animal welfare legislation for the care and use of animals and approved by the Third Military Medical University Chongqing, China. We used minimal number of animals and tried to minimize their suffering. Male Sprague-Dawley (SD) rats, weighing 275–300 g, were housed in specific pathogen-free (SPF) condition, with free access to water and food. After the rats were anesthetized with an i.p. injection of chloral hydrate (100 mg/kg body weight) and placed in a stereotaxic apparatus, a guide cannula (AG-8; Eicom, Tokyo, Japan) was implanted into the third ventricle (coordinates from Bregma: 3.9 mm posterior, 0.9 mm lateral, 8.4 mm below skull surface angled at 5° to vertical toward the midline), and fixed to the skull with dental cement and small screws, according to the coordinates provided by Klippel’s atlas. During the 1-week postoperative recovery period, rats were acclimated to handling and the experimental cage used for drug administration. We cannulated a total of 130 rats, of which two were excluded from the study because of cannula misplacement.

### Hypoxia

Rats of the hypoxia group were placed in a hypobaric chamber simulating an elevation of 3700 m and 6000 m for different times, and then immediately sacrificed.

### Thirst Studies

**Figure 1:**
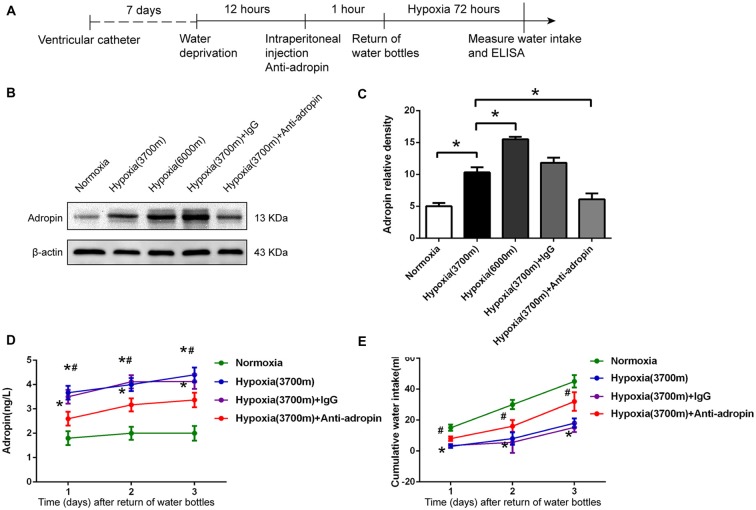
**Anti-dipsogenic effects were related to increased adropin under hypoxia in rats. (A)** The experimental design to study the mechanism of central anti-dipsogenic effects (Figures 1, 2). **(B)** Adropin protein in circumventricular organs (CVOs) nuclei was down-regulated 25 h after injection of 10 μl adropin antibody into the third ventricle after overnight water deprivation-induced drinking in rats. **(C)** Relative protein expression was calculated (*n* = 6 for each group, *P < 0.05).** (D)** The expression of serum adropin was enhanced under hypoxia (3700 m and 6000 m) at 1, 2 and 3 days after overnight water deprivation-induced drinking in rats (*n* = 8 for each group; *P < 0.05 vs. normoxia group; ^#^*P* < 0.05 vs. 3700 m hypoxia group).** (E)** Hypoxia induced anti-dipsogenic effects was alleviated after administration of 10 μl rat source special adropin antibody under hypoxia (3700 m) after overnight water deprivation-induced drinking in rats (IgG as a control, *n* = 8 for each group; *P < 0.05 vs. normoxia group; ^#^*P* < 0.05 vs. 3700 m hypoxia group).

Drinking water was not provided to the rats for 12 h before the experiment. Normoxic and hypoxic animals were placed in an environment of sea level or a hypobaric chamber. To ensure the accuracy of measuring water consumption, we filled the bottles with water and completely eliminated any gas in the bottles. The consumption of water was measured through the original water weight minus the current water weight after hypoxia. Experimental design see Figures 1A, 3A, 5A.

**Figure 2:**
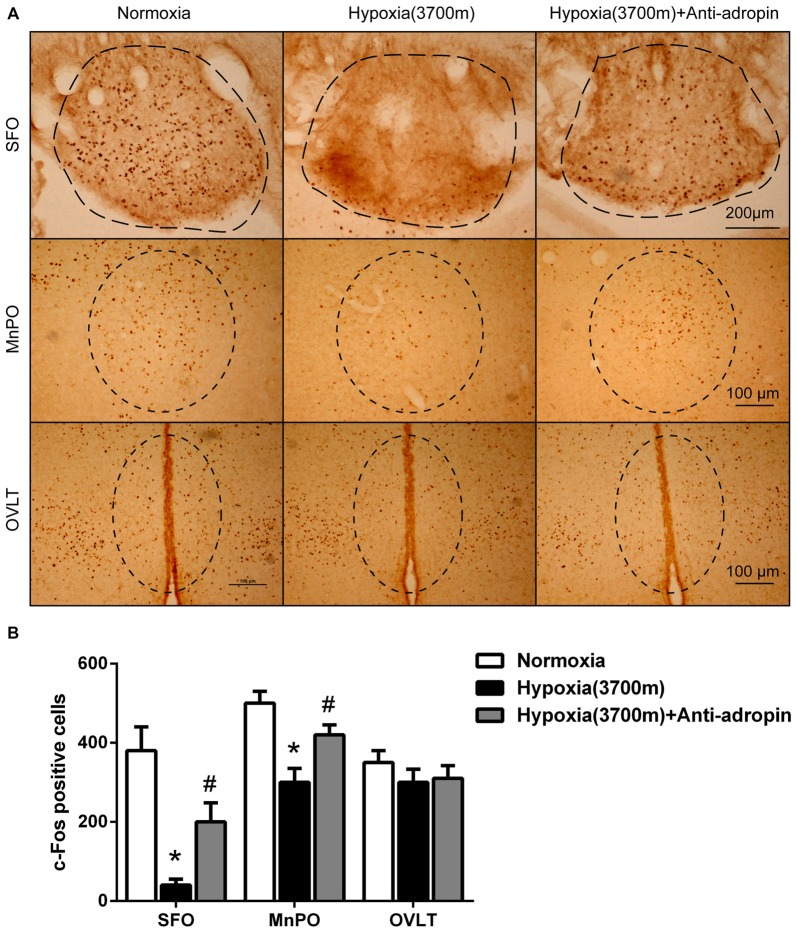
**Suppression of c-Fos under hypoxia was relieved when adropin was neutralized in CVO nuclei of rats. (A)** The reduction of c-Fos positive cells (visualized as brown staining) in Subfornical organ (SFO) and Median preoptic nucleus (MnPO) regions under hypoxia (3700 m) was recovered 25 h after administration of 10 μl adropin antibody in the third ventricle after overnight water deprivation-induced drinking in rats, Scale bar, SFO:200 μm, MnPO, OVLT: 100 μm. **(B)** Quantification of c-Fos positive cells (three rats for each group; *P < 0.05 vs. normoxia group, ^#^*P* < 0.05 vs. 3700 m hypoxia group).

**Figure 3:**
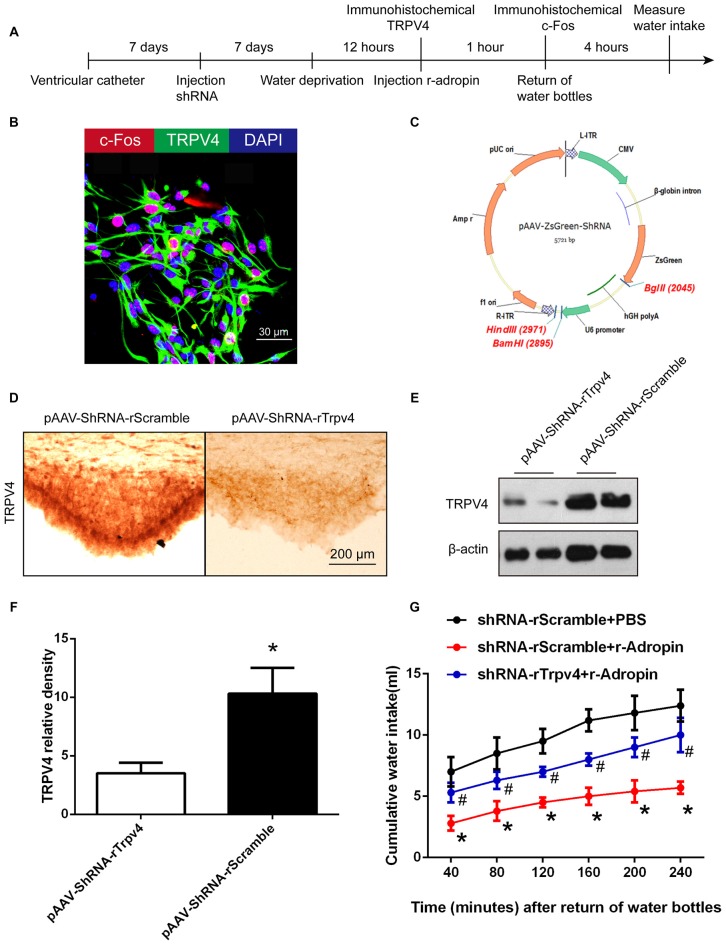
**Central administration of recombinant adropin mimicked anti-dipsogenic effects by activating TRPV4 neurons under normoxic condition in SFO. (A)** The experimental design to study the mechanism of central anti-dipsogenic effects (Figures 3, 4). **(B)** TRPV4 (green) and c-Fos (red) within the same cells in SFO of wild-type rats after water deprivation for 12 h. The right panel shows a magnified view illustrating the overlap between c-Fos and TRPV4 positive signals. Nuclei were counterstained with DAPI (blue). Scale bar, 30 μm. **(C)** Construction of pAAV-ZsGreen-ShRNA cloning vector for S. *thermophiles*. **(D)** TRPV4 (visualized as brown staining) was down-regulated in SFO 7 days after injection of Trpv4 shRNA into the third ventricle. Scrambled shRNA was used as a control. Scale bar, 200 μm. **(E)** TRPV4 in SFO was detected by western blot. **(F)** Relative protein expression was calculated (*n* = 6 for each group, *P < 0.05). **(G)** Central administration of recombinant adropin (3 nM) inhibited water intake after 1 h and the inhibitory action of adropin was blocked by TRPV4 shRNA after 4 h in overnight water-deprived rats (*n* = 8 for each group; *P < 0.05 vs. control group; ^#^*P* < 0.05 vs. recombinant adropin group).

**Figure 4:**
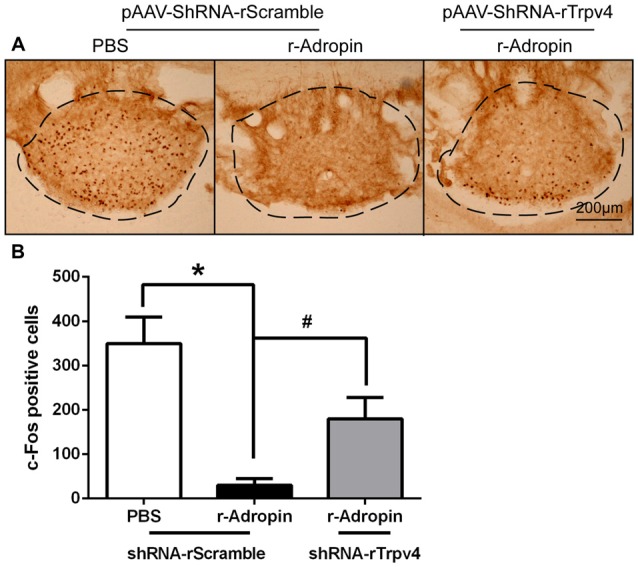
**Central administration of recombinant adropin reduced c-Fos positive cells via TRPV4 signaling under normoxic condition in SFO. (A)** c-Fos positive cells (visualized as brown staining) in SFO decreased 1 h after central administration of 3 nM recombinant adropin in overnight water deprivation-induced drinking in rats. The adropin-induced c-Fos reduction was reversed by TRPV4 shRNA, Scale bar, 200 μm. **(B)** Quantification of c-Fos positive cells (three rats for each group; *P < 0.05, ^#^*P* < 0.05).

**Figure 5:**
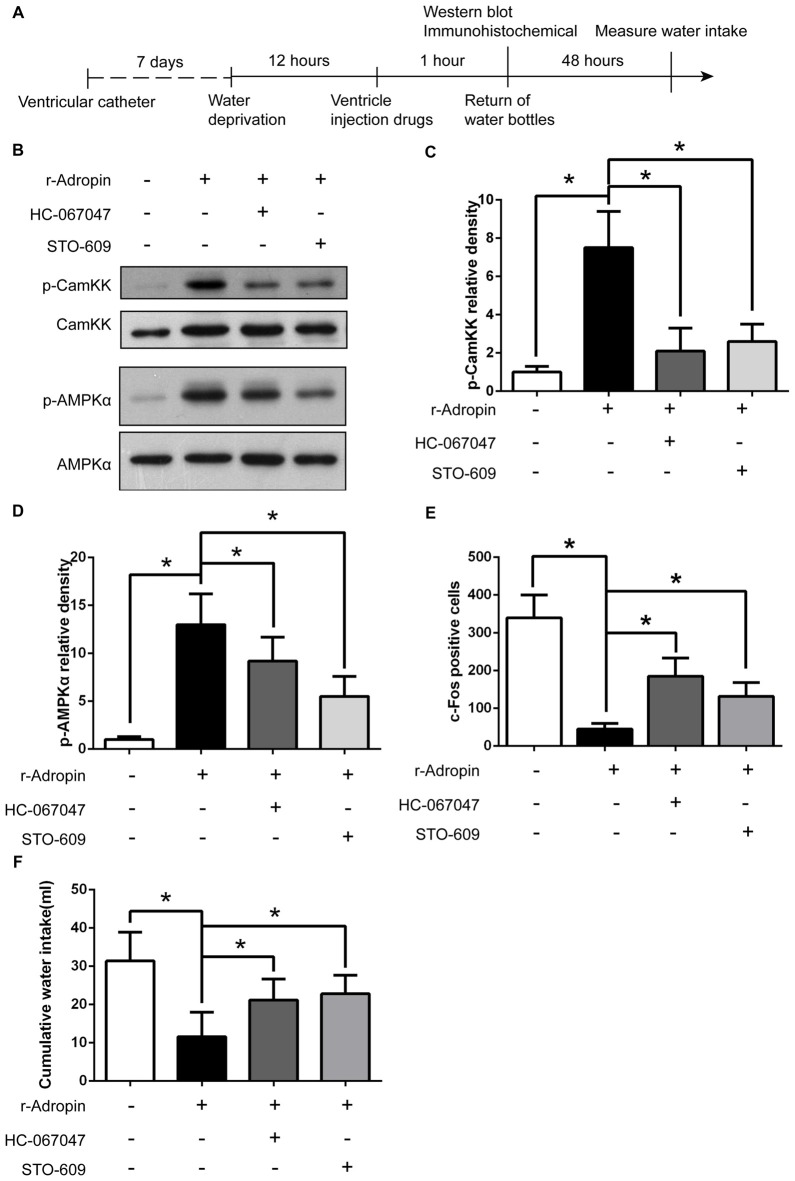
**The anti-dipsogenic effects of recombinant adropin was regulated by TRPV4-Camkk-AMPK pathway under normoxic condition in SFO. (A)** The experimental design to study the mechanism. **(B)** p-Camkk and p-AMPK in SFO were detected 1 h after central injection of 3 nM recombinant adropin or 1 mM HC067047 or 1.5 mM STO-609 (a Camkk inhibitor) in overnight water-deprived rats. **(C,D)** Relative p-Camkk and p-AMPK protein expression was calculated (*n* = 6 for each group; *P < 0.05). **(E)** c-Fos positive cells in SFO was counted 1 h after central injection of drugs in overnight water-deprived rats (*n* = 3 for each group; *P < 0.05). **(F)** Cumulative water intake within 48 h after central injection of drugs in overnight water-deprived rats (*n* = 8 for each group; *P < 0.05).

### Enzyme-Linked Immunosorbent Assay

Enzyme-linked immunosorbent assays (ELISA) were performed as previously reported (Yang C. et al., 2016). After centrifugation at 3000 g for 20 min, serum adropin was quantified in the supernatant by ELISA according to the manufacturer’s instructions (Cloud-Clone Corp, Carlsbad, CA, USA). The optical absorbance was measured at 450 nm with a BioTek Elx800 microplate reader. Proteins in the serum were quantified by interpolation of standard curves generated from the recombinant adropin provided by the vendor.

### Western Blotting

After the rats were sacrificed, the brain was removed rapidly, and the SFO area was carefully separated and dissected under the anatomical microscope, RIPA cell lysate (Beyotime, China) was added, and the supernatant was obtained after centrifugation for 3000 times. Total protein was extracted and concentrations were measured using a bicinchoninic acid (BCA) protein assay. The following antibodies were used: anti-adropin (1:500, Cloud-Clone Corp, Carlsbad, CA, USA), anti-TRPV4 (1:500, Abcam), anti-CamKK (1:500, Abcam), anti-p-CamKK (1:500, Abcam), anti-AMPKα (1:1000, Cell Signaling Cambridge, UK), anti-p-AMPKα (1:1000, Cell Signaling Cambridge, UK) and anti-β-actin (1:1000, Santa Cruz Biotechnologies, Dallas, TX, USA). The immunoreactive bands were visualized using enhanced chemiluminescence (Amersham Biosciences, Arlington Heights, IL, USA), according to the manufacturer’s instructions. The expression bands of target proteins were detected on a bio-imaging system (VersaDoc MP 4000; Bio-Rad, Hercules, CA, USA) and ImageJ software was used to analyze the densitometric values. The housekeeping protein β-actin was used as an internal control.

### Drugs and shRNA

In the different experiments, 10 μl adropin antibody (Cloud-Clone Corp, Carlsbad, CA, USA), 3 nM rat recombinant adropin (Cloud-Clone Corp, Carlsbad, CA, USA), 1 mg/ml normal rat IgG (Beyotime Biotechnology), 2.28 × 10^12^ titer adeno-associated virus (AVV) packed pAAV-ZsGreen-shRNA (Agilent, BioWit Technologies), 1 mM HC067047, and 1.5 mM STO-609 were microinjected into the third ventricle using a microinjection cannula inserted into the guide cannula, respectively. The microinjection cannula was connected via a polyethylene tube to a micro syringe containing different drug solutions at a total volume of 50 μl. The control groups received the same volume of sterile saline at the same time. Five minutes after the injection, the microinjection cannula was switched to a dummy cannula. The presence of TRPV4 shRNA gene in recombinant viral DNA was verified by polymerase chain reaction (data not shown). The forward primer sequence was 5′-GATCCGCTGGATGAATGCCCTTTACTTTCAAGAGAAGTAAAGGGCATTCATCCAGCTTTTTTAGATCTA-3′ and the reverse primer sequence was 5′-AGCTTAGATCTAAAAAAGCTGGATGAATGCCCTTTACTTCTCTTGAA AGTAAAGGGCATTCATCCAGCG-3′. Scramble shRNA sequence was used as the control group, Plasmid vector design see Figure 3C the sequence is as follows: forward, 5′-AACTTTCTCCGAACGTGTCACGTTTCAAGAGAACGTGACACGTTCGGAGAATTTTTTC-3′, and reverse, 3′-TTGAAAGAGGCTTGCACAGTGCAAAGTTCTCTTGCACTGTGCAAGCCTCTTAAAAAAGAGCT-5′.

### Immunohistochemistry

Cells and brain tissue samples were fixed in 4% formaldehyde, dehydrated in a 30% sucrose solution, and sectioned into 20-μm-thick sections using Leica Microsystems Nussloch GmbH (D-69226, Germany). After blocking with 10% normal goat serum, the samples were permeabilized using 0.1% Triton-X 100, and incubated with various primary antibodies, including anti-adropin (1:200), anti-c-Fos (1:200) and anti-TRPV4 (1:200; Abcam, Cambridge, UK). After washing, samples were probed with the appropriate secondary antibodies (Jackson Immunoresearch, West Grove, PA, USA). Micrographs were selected and captured using a laser confocal microscope, and analyzed using MagnaFire SP 2.1B software (Olympus, Melville, NY, USA).

### Statistical Analysis

Data were analyzed by SPSS13.0 software and presented as means ± SD. A one-way analysis of variance (ANOVA) with repeated measures was used to estimate the significance of water intake at different times. The Student’s *t*-test and ANOVA were used for two or more group comparisons, respectively. The results were statistically significant at *p* < 0.05.

## Results

### Water Intake was Decreased and Adropin was Simultaneously Elevated during Early Hypoxia in Rats

We verified the anti-dipsogenic effects at moderate (3700 m) and severe hypoxia (6000 m). As previously reported (Jones et al., 1981a,b; Yang F. et al., 2016), our data also showed that water consumption in the 3700 m hypoxia group decreased > 40% at 1 day after 12 h water restriction as compared to the normoxia group, and the inhibition effect continued for at least 3 days (Figure 1E). We also tested adropin, which functions as a vascular protective factor in Type 2 diabetes patients and an inhibitor in the brain center of water drinking through an orphan G protein-coupled receptor 19 (GPR19; Topuz et al., 2013; Hu and Chen, 2016; Stein et al., 2016). Interestingly, our results showed that serum adropin was elevated under 3700 m hypoxia, and further increased in the 6000 m hypoxia group as compared to the moderate group (Figure 1D). In addition, we reconfirmed that adropin production was also enhanced in the CVOs of brain under hypoxia (Figures 1B,C). These data suggested that the hypoxia-induced adropin could be associated with anti-dipsogenic effects by affecting the drinking center in the brain.

### Thirst Neurons in CVOs were Inhibited Under Hypoxia

We hypothesized that the hypoxia-induced anti-dipsogenic effects were associated with inhibition of CVOs, which regulate primary central drinking, including SFO, MnPO and OVLT nuclei. If these CVO neurons function as key cellular switches in the circuit that drives water consumption, then their inhibition should trigger anti-dipsogenic effects. To investigate the status of CVOs under hypoxia, c-Fos was used as a marker for neuronal activation. Approximately 30% of cells in the SFO of 48 h water-restricted mice were c-Fos positive, but no c-Fos labeling was detected after water satiety (Oka et al., 2015). As expected, immunohistochemical data revealed that c-Fos positive cells were broadly decreased in SFO and MnPO on the first day of hypoxia, with partial recovery on the third day (Figures 2A,B), suggesting that the CVO neurons were inhibited under hypoxia in rats, which may be a key to anti-dipsogenic effects.

### Hypoxia-Induced Increased Adropin Could Inhibit Water Intake and Neuronal Activity in CVOs of Rats

Adropin is a type of secretory protein, whose extracellular content is higher than intracellular (Li et al., 2016; Shahjouei et al., 2016). Using a specific scavenger adropin antibody, we neutralized and down-regulated adropin by stereotaxic injection in CVOs. The adropin antigen binding site was shrouded (Cys34-Pro76) so that adropin was decreased to 43% by western blot (Figures 1C,D) and its biological effects might have been disabled. After adropin protein was neutralized, we also observed that water consumption (Figure 1E) and c-Fos positive cells (Figure 2A) were significantly increased in CVOs for 1–3 days under 3700 m hypoxia.

To verify the anti-dipsogenic effects of adropin under normoxia, we injected recombinant adropin into the third ventricle, which is close to CVOs. Consistent with previous reports, recombinant adropin reduced water intake in normoxia (mimicking anti-dipsogenic effects) similar to that in hypoxia (Figure 3E). These results strongly suggested that increased adropin in hypoxia is vital for anti-dipsogenic effects.

### Adropin Induced Anti-Dipsogenic Effects through TRPV4 Calcium Channel in SFO

Some reports suggested that adropin could combine with membrane receptors that modulate biological function, such as G-protein-coupled receptors (Stein et al., 2016). Transient receptor potential vanilloid 4 (TRPV4), a non-selective cation channel permeable to Ca^2+^, was initially characterized as an osmosensor in CVOs (Liedtke and Friedman, 2003; Noda and Sakuta, 2013). Our previous study suggested that TRPV4 inhibitor could alleviate anti-dipsogenic effects and restore water consumption under 5000 m hypoxia (Yang F. et al., 2016). In this study, we hypothesized that if activation of TRPV4 neurons by adropin extinguished thirst, then the effect should be specific for the restriction of water consumption. Thus, to explore whether TRPV4 is involved in anti-dipsogenic effects on adropin, recombination adropin protein inhibitory rate see Supplementary Figure S1 was microinjected into the third ventricle and TRPV4 was knocked down by shRNA in SFO of rats (Figures 3D–F). Interestingly, TRPV4 and c-Fos co-location to SFO (Figure 3B) and the restrained water intake after adropin was partially reversed after knock-down of TRPV4 (Figure 3G). In addition, administration of recombinant adropin did not decrease c-Fos positive cells in SFO of TRPV4 knock-down rats as compared to scramble control (Figures 4A,B). These results suggested that adropin induced anti-dipsogenic effects through TRPV4 in SFO.

### Adropin Induced Anti-Dipsogenic Effects through TRPV4-Camkk-AMPK Signaling in SFO

Anti-dipsogenic effects of adropin was inhibited after HC-067047 (a TRPV4 inhibitor) administration (Figure 5F). This further proved that adropin produced anti-dipsogenic effects through TRPV4 expression, but the underlying molecular mechanism was unclear. Our previous study demonstrated that TRPV4 opened with Ca^2+^ influx when cells were exposed to hypoxia (Yang F. et al., 2016). In the present study, pho-calmodulin-dependent protein kinase kinase (Camkk) and pho-AMP-activated protein kinase (AMPK) α subunit in CVOs were up-regulated after adropin administration (Figures 5B–D). Moreover, both TRPV4 inhibitor HC-067047 and Camkk inhibitor STO-609 could decrease p-AMPKα (Figures 5B–D), and increased water intake (Figure 5F) and c-Fos positive cells in SFO (Figure 5E). These results suggested that TRPV4-Camkk-AMPK signaling participated in adropin-induced anti-dipsogenic effects in SFO in rats.

## Discussion

Identifying the reason for reduction in thirst and water consumption under hypoxia will facilitate understanding of acclimatization in early plateau migratory population (Yang F. et al., 2016). Adropin showed inhibitory effects on thirst centers through the orphan GPR19 (Stein et al., 2016). In the present study, adropin expression under hypoxia and its influence on the CNS, including CVOs was studied (Miselis, 1981; McKinley et al., 2003). The following conclusions were drawn: first, increased expression of adropin under hypoxia is associated with less water intake (anti-dipsogenic effects) regulated by thirst-related nuclei in the CNS. Second, adropin exerts anti-dipsogenic effects through TRPV4 in CVOs. Third, adropin-induced anti-dipsogenic effects is achieved by the TRPV4 downstream calcium-mediated Camkk-AMPK pathway.

The body’s fluid homeostasis is adjusted through sodium and water metabolism (Noda and Sakuta, 2013; Segar, 2017; Langston, 2017). Once destroyed, sensitive areas of the brain can perceive this change and then initiate the related target intake behavior. For example, salt-deprived animals consume salt so voraciously that their salt levels are higher than normal (Daniels and Fluharty, 2004). Similarly, water-deprived animals have increased motivation of drinking water (Stricker and Sved, 2000; McKinley and Johnson, 2004). Previous studies found that CVOs are the closest in relation with drinking water outside the BBB, such as SFO, MnPO and OVLT, which constitute nerve fibers network and contribute to the stability of osmotic pressure (Miselis, 1981; Ho et al., 2007). As compared to high urea or sugar, high osmotic pressure easily affects the CVO neurons, and ultimately retro-regulates animal’s thirst perception and water consumption to reduce plasma osmotic pressure (Noda and Sakuta, 2013; Oka et al., 2015).

The CVOs sense the osmotic pressure through their cation channels on the cell membrane, which generally fall under two categories: one type is sodium sensor channels, including Nax and NALCN (Goldin et al., 2000; Watanabe et al., 2000); while the other type is calcium ion channels delegated with TRPV1 and TRPV4 (Ciura and Bourque, 2006; Benfenati et al., 2011). Both work independently and cooperate with each other under thirst conditions (Noda and Sakuta, 2013), but the mechanism remains unclear. TRPV4 is a transient calcium ion channel, which exists in cell membranes and endoplasmic reticulum, and is activated by temperature, acid and hormone stimuli (Liedtke et al., 2000; Liedtke and Friedman, 2003; Mizuno et al., 2003). In the present study, a new small molecule polypeptide called adropin, which is composed of 76 amino acids, was found to mimic anti-dipsogenic effects after intracranial injection. Interestingly, the anti-dipsogenic effects of adropin was alleviated when TRPV4 was knocked down in SFO. Our previous study had found that relying on the heme-oxygenase 2, hypoxia could open TRPV4 channels of SFO neurons directly and rapidly, and decreased thirst in rats, which indicated that TRPV4 is key in hypoxia-induced anti-dipsogenic effects (Yang F. et al., 2016). These results suggested that hypoxia stress could regulate water consumption through direct and indirect ways. Low partial pressure of oxygen opened TRPV4 due to lack of BBB in CVOs, while hypoxia increased adropin expression, which passed through BBB and activated TRPV4.

Adropin is a secreted peptide mainly produced in the liver and CNS (Shahjouei et al., 2016). Stein et al. (2016) first reported that the central effect of adropin led to reduction in water consumption via GPR19. In the adult mouse, GPR19 showed high levels of transcription in several regions of the brain, including the olfactory bulb, the hippocampus and hypothalamic (Hoffmeister-Ullerich et al., 2004). These nuclei are involved in a variety of biological effects, leading to the central injection of adropin can change the animal’s drinking water, eating behavior and arterial blood pressure (Stein et al., 2016). The regulation of Adropin in the CNS may involve multiple receptors, not only through GPR19, but also by TRPV4. This implied that adropin may be a signaling peptide that participated in liver-brain crosstalk. Hypoxia stress may magnify these effects because adropin is up-regulated and cerebrovascular endothelial permeability is increased (Yang C. et al., 2016). In fact, adropin was significantly increased in the first day of hypoxia, and was maintained at a high level until the third day. In addition, recombinant adropin reduced water intake and imitated hypoxia-induced anti-dipsogenic effects in normoxia, but neutralizing adropin could restore thirst in hypoxia. These results confirmed that increased adropin is important for hypoxia-induced anti-dipsogenic effects.

Adropin activated TRPV4 and reduced water intake, but TRPV4 inhibitor restored thirst. Depending on the TRPV4 open and extracellular calcium influx, adropin increased the phosphorylation of calcium/calmodulin-dependent protein kinase kinase (CamKK) and AMP-activated protein kinase α (AMPKα). AMPK is a highly conserved serine/threonine protein kinase that regulates energy homeostasis and metabolic stress, and exists in all eukaryotic cells as heterotrimeric complexes comprising catalytic α-subunits and regulatory β- and γ-subunits. Phosphorylation of the Thr172 residue in the α-subunit is important for optimal AMPK activity (Choi et al., 2016). So adropin increased AMPKα phosphorylation, which is conducive to TRPV4 neuronal excitation in CVOs. Cota et al. (2006) found that AMPK phosphorylation participated in the regulation of feeding behavior in hypothalamus, while our data suggested that it is related to drinking behavior in CVOs.

Adropin significantly activated TRPV4 neuronal excitation, while the activity of SFO declined due to the reduction of c-Fos positive cells. This contradiction could be explained by two assumptions: First, activation of TRPV4 neurons by adropin restrained water intake, and adropin might suppress other ion channels promoting water intake in CVOs, such as TRPV1 and NALCN. Second, activation of TRPV4 neurons induced by adropin could inhibit other neurons promoting water intake through the nerve fibers projection in CVOs. Notably, the expression of adropin increased and TRPV4 neurons were activated, which were directly involved in hypoxia-induced anti-dipsogenic effects.

In summary, we found that a new small molecule peptide adropin showed anti-dipsogenic effects under hypoxia via TRPV4-CamKK-AMPK signaling in CVOs of rats. This study enhances the understanding of water sodium balance and pathological rehydration in early hypoxia.

## Author Contributions

FY, LZ, XQ, DW, W-JH, ZT, JY and Q-YH conceived the experiments, reviewed the manuscript. FY, LZ and DW conducted the experiments and XQ analyzed the results. Q-YH wrote the manuscript.

## Conflict of Interest Statement

The authors declare that the research was conducted in the absence of any commercial or financial relationships that could be construed as a potential conflict of interest.
