# Supplementary figures and images for: Adropin Is a Key Mediator of Hypoxia Induced Anti-Dipsogenic Effects via TRPV4-CamKK-AMPK Signaling in the Circumventricular Organs of Rats
Source: Front Mol Neurosci. 2017 Apr 20;10:105. doi: 10.3389/fnmol.2017.00105 (PMC5397471; doi:10.3389/fnmol.2017.00105)

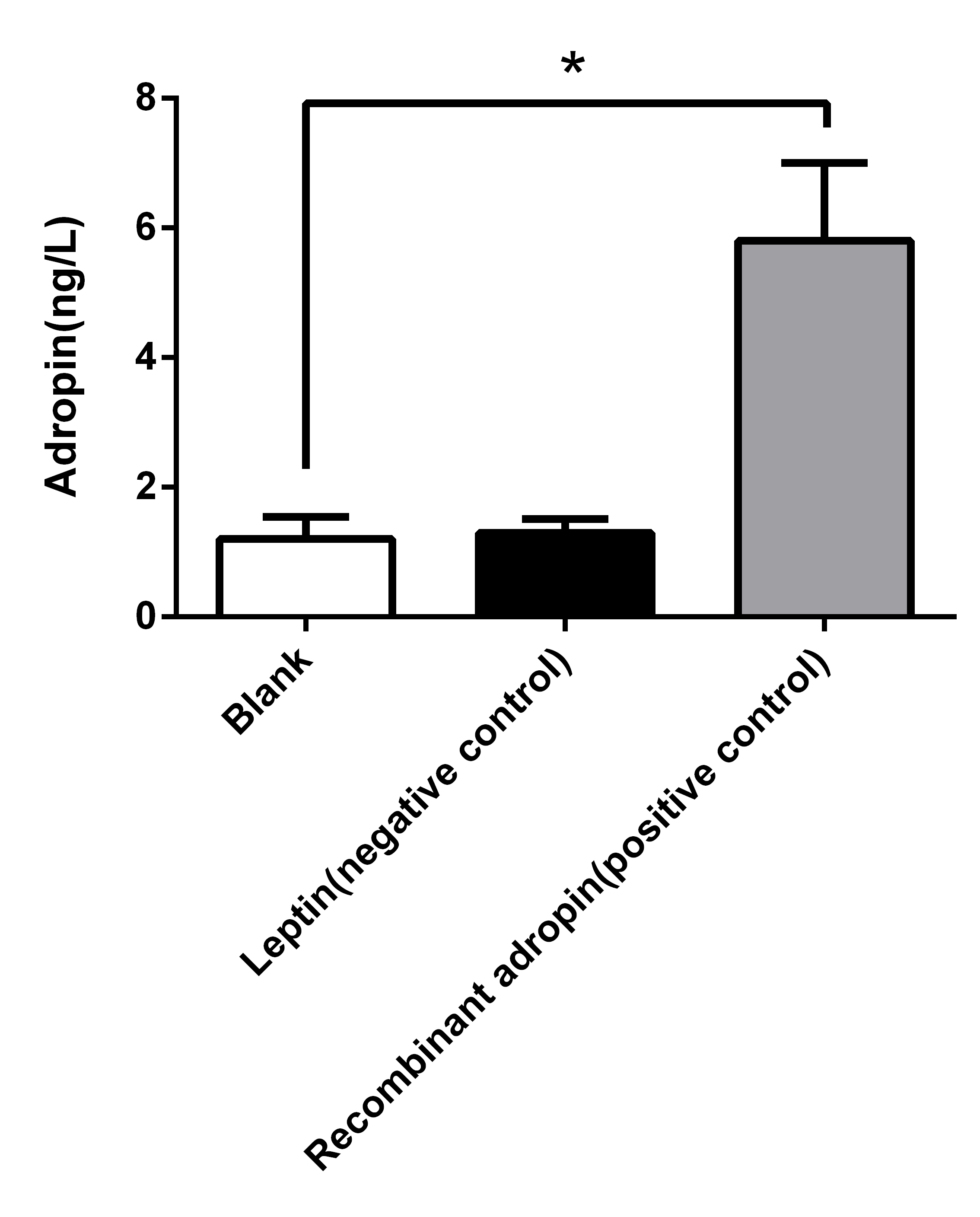

Supplement: Supplementary file 1 [file Image_1.tif]
